# Supplementary figures and images for: The outcomes of three different techniques of coronary artery bypass grafting: On-pump arrested heart, on-pump beating heart, and off-pump
Source: PLoS One. 2023 May 31;18(5):e0286510. doi: 10.1371/journal.pone.0286510 (PMC10231783; doi:10.1371/journal.pone.0286510)

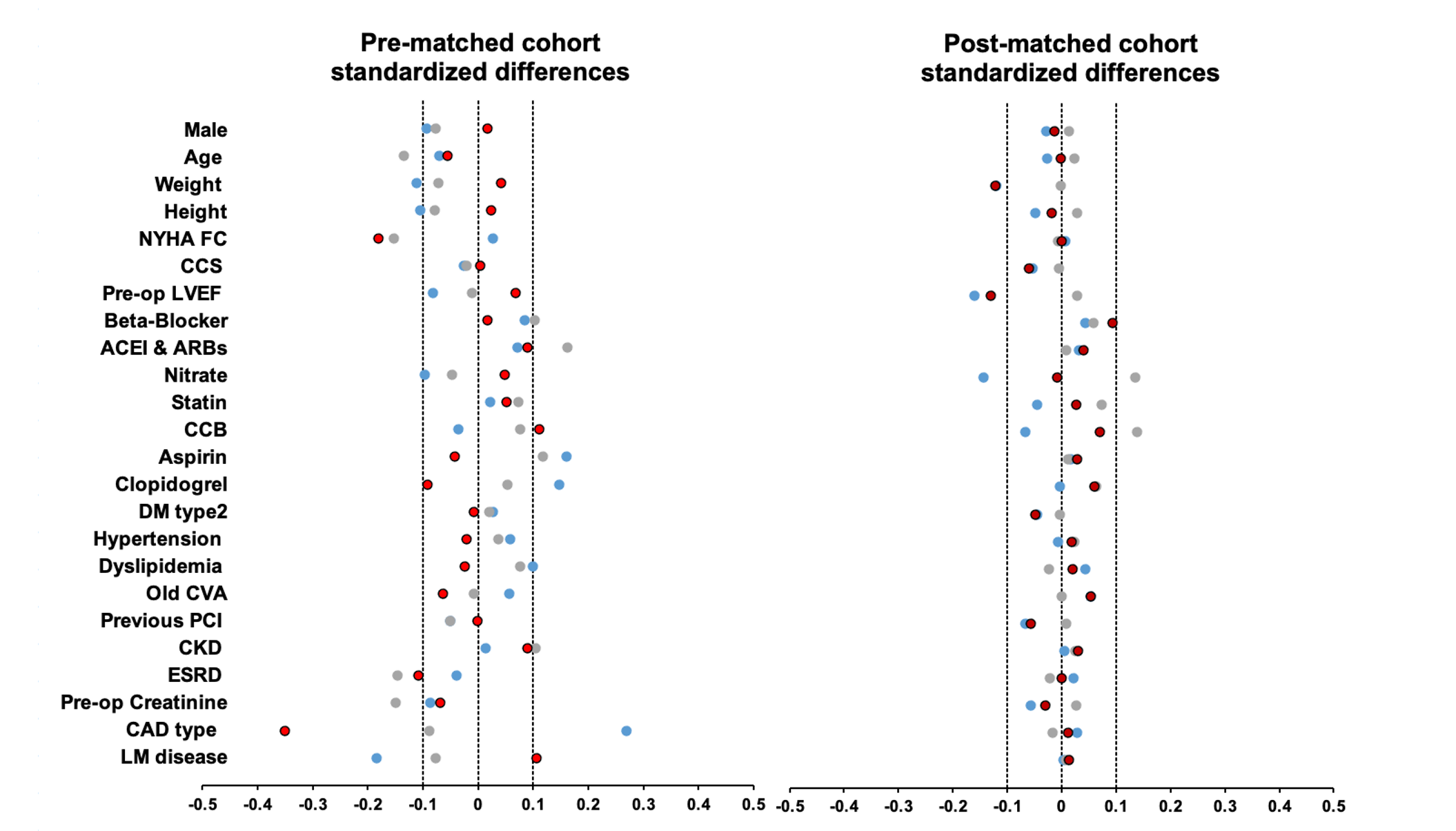

Supplement: S1 Fig — NYHA FC, New York Heart Association functional classification; CCS, Canadian Cardiovascular Society Classification; Pre-op, Pre-operative; LVEF, Left ventricular ejection fraction; ACEI/ARBs, Angiotensin-converting enzyme inhibitor drugs/Angiotensin receptor blockers drugs; CCB, Calcium channel blocker; DM, Diabetes Miletus; CVA, Cerebrovascular disease; PCI, percutaneous cardiac intervention; CKD, Chronic kidney disease; ESRD, End-stage renal disease; CAD, Coronary artery disease; LM, Left main. (TIF) [file pone.0286510.s001.tif]

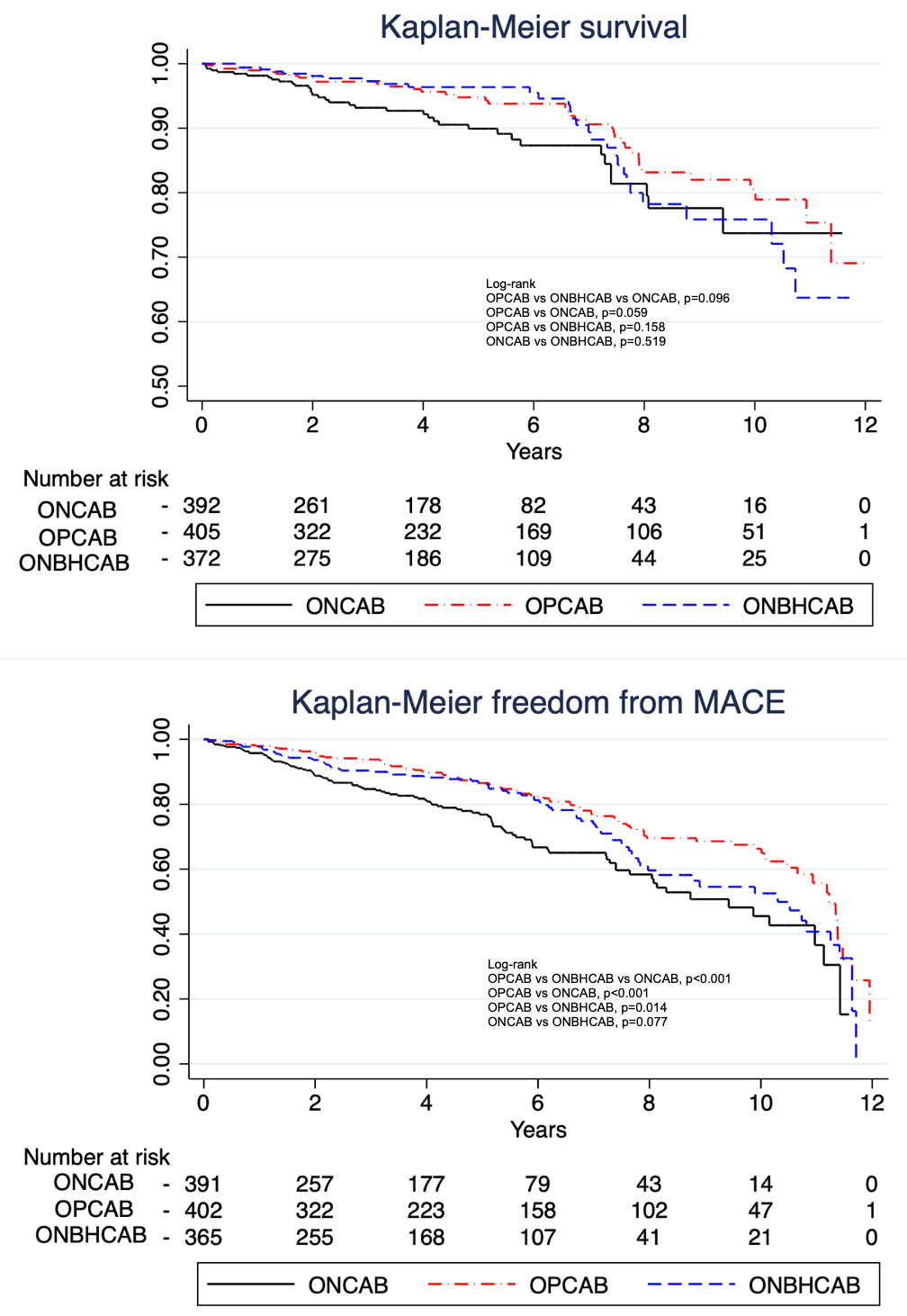

Supplement: S2 Fig — OPCAB, Off-pump coronary artery bypass; ONBHCAB, On-pump beating heart coronary artery bypass; ONCAB, On-pump arrested heart coronary artery bypass; MACE, Major adverse cardiovascular events: The composite of total death, myocardial infarction, coronary revascularization, stroke, and heart failure. (TIF) [file pone.0286510.s002.tif]
