# Supplementary material for: The outcomes of three different techniques of coronary artery bypass grafting: On-pump arrested heart, on-pump beating heart, and off-pump
Source: PLoS One. 2023 May 31;18(5):e0286510. doi: 10.1371/journal.pone.0286510 (PMC10231783; doi:10.1371/journal.pone.0286510)
Supplement: S1 Table — (DOCX) [file pone.0286510.s003.docx]

**S1 Table. Standardized differences data between pre-matched and post-matched of patients’ baseline characteristics.**

|  | Pre-Match STD | | | |  | Post-Match STD | | | |
| --- | --- | --- | --- | --- | --- | --- | --- | --- | --- |
| Variable | p | OPCAB vs ONCAB | OPCAB vs ONBHCAB | ONCAB vs ONBHCAB |  | p | OPCAB vs ONCAB | OPCAB vs ONBHCAB | ONCAB vs ONBHCAB |
| Male | 0.154 | -0.094 | -0.077 | 0.017 |  | 0.920 | -0.028 | 0.014 | -0.014 |
| Age | 0.042 | -0.070 | -0.135 | -0.055 |  | 0.903 | -0.027 | 0.024 | -0.001 |
| Weight | 0.101 | -0.112 | -0.071 | 0.042 |  | 0.116 | -0.120 | -0.001 | -0.122 |
| Height | 0.116 | -0.105 | -0.079 | 0.023 |  | 0.772 | -0.049 | 0.029 | -0.018 |
| NYHA FC | 0.020 | 0.027 | -0.153 | -0.181 |  | 0.994 | 0.006 | -0.006 | 0.000 |
| CCS | 0.011 | -0.025 | -0.021 | 0.004 |  | 0.982 | -0.053 | -0.005 | -0.060 |
| LVEF | 0.336 | -0.082 | -0.010 | 0.069 |  | 0.053 | -0.161 | 0.029 | -0.131 |
| Beta-Blocker | 0.100 | 0.084 | 0.103 | 0.018 |  | 0.311 | 0.044 | 0.058 | 0.093 |
| ACEI & ARBs | 0.008 | 0.072 | 0.163 | 0.090 |  | 0.823 | 0.031 | 0.009 | 0.040 |
| Nitrate | 0.208 | -0.096 | -0.047 | 0.049 |  | 0.058 | -0.144 | 0.135 | -0.009 |
| Statin | 0.358 | 0.022 | 0.074 | 0.052 |  | 0.542 | -0.045 | 0.073 | 0.027 |
| CCB | 0.155 | -0.035 | 0.076 | 0.112 |  | 0.122 | -0.067 | 0.139 | 0.071 |
| Aspirin | 0.006 | 0.160 | 0.118 | -0.042 |  | 0.918 | 0.017 | 0.011 | 0.028 |
| Clopidogrel | 0.027 | 0.147 | 0.054 | -0.092 |  | 0.575 | -0.004 | 0.064 | 0.060 |
| DM type2 | 0.857 | 0.027 | 0.020 | -0.007 |  | 0.311 | -0.045 | -0.004 | -0.049 |
| Hypertension | 0.533 | 0.058 | 0.037 | -0.020 |  | 0.823 | -0.006 | 0.024 | 0.018 |
| Dyslipidemia | 0.131 | 0.100 | 0.076 | -0.024 |  | 0.058 | 0.044 | -0.024 | 0..020 |
| Old CVA | 0.538 | 0.056 | -0.008 | -0.064 |  | 0.542 | 0.054 | 0.000 | 0.054 |
| Previous PCI | 0.505 | -0.050 | -0.051 | -0.001 |  | 0.122 | -0.066 | 0.009 | -0.057 |
| CKD | 0.118 | 0.014 | 0.104 | 0.090 |  | 0.918 | 0.005 | 0.025 | 0.030 |
| ESRD | 0.015 | -0.039 | -0.146 | -0.108 |  | 0.575 | 0.022 | -0.022 | 0.000 |
| Pre-op Cr | 0.013 | -0.086 | -0.149 | -0.068 |  | 0.311 | -0.057 | 0.027 | -0.030 |
| CAD type | 0.000 | 0.269 | -0.088 | -0.350 |  | 0.823 | 0.028 | -0.017 | 0.011 |
| LM disease | 0.004 | -0.184 | -0.077 | 0.106 |  | 0.985 | 0.004 | 0.009 | 0.013 |
| STD, Standardize deviation; NYHA FC, New York Heart Association functional classification; CCS, Canadian Cardiovascular Society Classification; Pre-op, Pre-operative; LVEF, Left ventricular ejection fraction; ACEI/ARBs, Angiotensin converting enzyme inhibitor drugs/Angiotensin receptor blockers drugs; CCB, Calcium channel blocker; DM, Diabetes Miletus; CVA, Cerebrovascular disease; PCI, percutaneous cardiac intervention; CKD, Chronic kidney disease; ESRD, End-stage renal disease; Cr, Creatinine; CAD, Coronary artery disease; LM, Left main.  Statistically significant at *p*<0.05 | | | | | | | | | |
