# Supplementary material for: The outcomes of three different techniques of coronary artery bypass grafting: On-pump arrested heart, on-pump beating heart, and off-pump
Source: PLoS One. 2023 May 31;18(5):e0286510. doi: 10.1371/journal.pone.0286510 (PMC10231783; doi:10.1371/journal.pone.0286510)
Supplement: S2 Table — (DOCX) [file pone.0286510.s004.docx]

**S2 Table. Surgeon detail.**

|  | All patients | | | | Post-matched with PS score | | | |
| --- | --- | --- | --- | --- | --- | --- | --- | --- |
| Variables | OPCAB  (n=517) | ONBHCAB  (n=595) | ONCAB  (n=916) | p | OPCAB  (n=443) | ONBHCAB  (n=443) | ONCAB  (n=443) | p |
| Surgeon, n (%) |  |  |  | <0.001 |  |  |  | <0.001 |
| -No.1 | 365 (70.6) | 53 (8.9) | 91 (9.9) |  | 316 (77.3) | 37 (8.4) | 52 (11.7) |  |
| -No.2 | 50 (9.7) | 511 (85.9) | 95 (10.4) |  | 40 (9.3) | 382 (86.2) | 46 (10.4) |  |
| -No.3 | 78 (15.9) | 12 (2.0) | 407 (44.4) |  | 68 (15.4) | 10 (2.3) | 202 (45.6) |  |
| -No.4 | 17 (3.3) | 13 (2.2) | 138 (15.1) |  | 13 (2.9) | 2 (0.5) | 69 (15.6) |  |
| -No.5 | 6 (1.2) | 4 (0.7) | 98 (10.7) |  | 5 (1.1) | 11 (2.5) | 39 (8.8) |  |
| -No.6 | 1 (1.9) | 2 (0.4) | 87 (9.5) |  | 1 (0.2) | 1 (0.2) | 35 (7.9) |  |
| OPCAB, Off-pump coronary artery bypass; ONBHCAB, On-pump beating heart coronary artery bypass; ONCAB, On-pump arrested heart coronary artery bypass; No., Number.  Statistically significant at *p*<0.05 | | | | | | | | |
