# Supplementary material for: The outcomes of three different techniques of coronary artery bypass grafting: On-pump arrested heart, on-pump beating heart, and off-pump
Source: PLoS One. 2023 May 31;18(5):e0286510. doi: 10.1371/journal.pone.0286510 (PMC10231783; doi:10.1371/journal.pone.0286510)
Supplement: S3 Table — (DOCX) [file pone.0286510.s005.docx]

**S3 Table. Postoperative cardiac enzyme and hemodynamic function analyzed by repeated measure mixed effects regression**

| Variables | Operation | | | | | | | | | p-value between three groups |
| --- | --- | --- | --- | --- | --- | --- | --- | --- | --- | --- |
|  | OPCAB  (n = 443) | | | ONBHCAB  (n = 443) | | | ONCAB  (n = 443) | | |  |
|  | Changed | 95 % CI | p | Changed | 95 % CI | p | Changed | 95 % CI | p |  |
| Cardiac enzyme | | | | | | | | | | |
| CK-MB | -18.5 | -22.3, -14.7 | <0.001 | -8.3 | -13.6, -3.0 | 0.002 | -20.2 | -25.3, -14.9 | <0.001 | <0.001 |
| cTnT | +13.2 | -70.7, 97.1 | 0.759 | +133.0 | 40.4, 225.5 | 0.005 | +3.6 | -82.1, 89.2 | 0.935 | 0.013 |
| Hemodynamic | | | | | | | | | | |
| CI | +0.22 | 0.16, 0.27 | <0.001 | +0.16 | 0.08, 0.21 | <0.001 | +0.20 | 0.14, 0.26 | <0.001 | 0.158 |
| MAP | -2.97 | -4.22, -1.72 | <0.001 | -3.66 | -5.01, -2.3 | 0.028 | -3.32 | -4.65, -1.99 | <0.001 | 0.744 |
| MPAP | -2.63 | -3.21, -2.05 | <0.001 | -1.96 | -2.56, -1.36 | <0.001 | -1.53 | -2.12, -0.94 | <0.001 | 0.026 |
| SVRI | -267.9 | -345.7, -190.1 | <0.001 | -187.8 | -271.2, -94.1 | <0.001 | -169.1 | -251.5, -86.6 | <0.001 | 0.743 |
| OPCAB, Off-pump coronary artery bypass; ONBHCAB, On-pump beating heart coronary artery bypass; ONCAB, On-pump arrested heart coronary artery bypass; Mean diff, Mean difference; CK-MB, creatine kinase-MB (mcg/L); cTnT, Cardiac Troponin T (ng/ml); CI, Cardiac Index (L/min.m2); MAP, Mean arterial pressure (mm Hg); MPAP, Mean pulmonary artery pressure (mm Hg); SVRI, Systemic vascular resistant index (dn.s.m2/cm5); Changed, Time interval changed.  Statistically significant at *p*<0.05 | | | | | | | | | | |
