# Supplementary material for: The outcomes of three different techniques of coronary artery bypass grafting: On-pump arrested heart, on-pump beating heart, and off-pump
Source: PLoS One. 2023 May 31;18(5):e0286510. doi: 10.1371/journal.pone.0286510 (PMC10231783; doi:10.1371/journal.pone.0286510)
Supplement: Attachment — Submitted filename: Response to reviewer.docx [file pone.0286510.s007.docx]

A rebuttal letter.

Dear Vincenzo Lionetti, Acedemic Editor

Thank you for giving us the opportunity to submit a revised draft of our manuscript titled “Outcomes of three different techniques of coronary artery bypass grafting: on-pump arrested heart, on-pump beating heart, and off-pump” to PLOS ONE. We appreciate the time and effort you and the reviewers have dedicated to providing valuable feedback on our manuscript. We are grateful to the reviewers for their insightful comments on our paper. We have been able to incorporate changes to reflect most of the suggestions provided by the reviewers.

Here is a point-by-point response to the reviewers’ comments and concerns.

**Comments to the Author**

**1. Is the manuscript technically sound, and do the data support the conclusions?

The manuscript must describe a technically sound piece of scientific research with data that supports the conclusions. Experiments must have been conducted rigorously, with appropriate controls, replication, and sample sizes. The conclusions must be drawn appropriately based on the data presented.**

**Reviewer #1: No**

**Reviewer #2: Yes**

Response: Thank you for pointing this out.

Our conclusion consists of

1. Postoperative ischemic injury (shown by the cardiac enzyme results); OPCAB implementation resulted in a lower occurrence of postoperative ischemic injury than ONCAB and ONBHCAB.
2. Postoperative hemodynamic function (shown by hemodynamic function results); No differences in postoperative hemodynamic function in all three techniques were observed.
3. Long-term outcomes (shown by the Survival curve and Free from MACE curve in the results); OPCAB respectively were preferable techniques beneficial for long-term outcomes.

That was all data drawn to support the conclusion.

**Comment from reviewer 1**

**Reviewer #1: Congratulations for the arguments that authors have selected.**

**Comment 1: Materials and Method, Reasoning behind the exclusion from off-pump to on-pump surgery?**

Response: Thank you for pointing this out.

1. Operative techniques were converted intraoperatively from OPCAB to on-pump CABG:

If the procedure was converted to ONBHCAB or ONCAB, it was an unplanned CABG procedure from the beginning. In other cases, the operation may have started as OPCAB but later, nearing the end of the procedure, the patient had to be converted, resulting in adverse outcomes from both OPCAB and on-CPB CABG.

2. Preoperative acute coronary syndrome, acute myocardial infarction:

Since this study requires the collection of cardiac enzymes to compare between groups, it may be difficult to interpret the rise in CK-MB and troponin levels if patients have an acute coronary syndrome or acute MI, as the increase in enzyme levels may not be solely attributed to postoperative values.

3. Preoperative shock:

The same reason as the acute MI, because this study needs to collect the hemodynamic data preoperative and postoperative to compare between groups, if patients have an unstable preoperative hemodynamic function, the postoperative value may be difficult to interpret.

4. Emergency CABG

In emergency cases, the PA catheter is usually not monitored, which means that hemodynamic values measured by the CCO monitor may not be collected. Additionally, emergency CABG surgeries may be complicated by severe acute MI or mechanical complications of MI, which often result in poor hemodynamic function."

**Comment 2: What kind of propensity matching algorithm did you use? After matching form smallest group (517 OPCAB) 443 remained.**

Response: Thank you for pointing this out.

A propensity score of multiple arms (3 groups), or the predicted probability of receiving ONCAB, OPCAB, and ONBHCAB, was calculated from the multinomial logistic regression model.

**Step 1**. we used multinomial logistic regression to create propensity scores for 3 groups. The variables included in the model for propensity score were described in the statistical analysis issue.

{command function in Stata 16.1}

Then we got the propensity score of three groups.

{table of the propensity score in each study group}

**Step 2.** Make the pattern that creates from the propensity score in each patient.

After finishing we get patterns like 513, 432, 423, 323 …..

**Step 3.** Match all three groups with the same pattern (in the same way as conventional propensity score matching, but use a pattern instead of score)

Example

- Pattern 125 had only one patient in group1, so this one patient from group 1 was removed from the study. (total 1 patient before matching, 0 patient after matching)
- Pattern 224 had two patients in group0, and four patients in group1&2 (a total of 10 patients before matching), after matching group 1&2 reduce to two patients each. (total 6 patients after matching)

The numbers before and after a match in each group.

|  | **before** | **after** |
| --- | --- | --- |
| **OPCAB** | **517** | **443** |
| **ONBHCAB** | **595** | **443** |
| **ONCAB** | **916** | **443** |

The reference for this method of propensity score matching was in the statistical analysis issue.

**Comment 3: Results, the quantity of grafts in groups was different?**

Response: Thank you for pointing this out.

We believe that the quantity of coronary anastomoses, as shown in Table 2, is a more important factor for long-term results than the number of graft conduits used. Therefore, we did not provide detailed information on the number of grafts used. However, in our CABG strategy, we typically use one graft conduit for each coronary main branch. When a connection to a sub-branch is needed, we usually connect the sequential graft conduit in the same main branch system. For example, we use LIMA to connect to the LAD and diagonal branch, radial artery to the OM branch and distal LCX, and SVG to the PD and/or PL."

**Comment 4: present P-value for survival differences.**

Response: Thank you for pointing this out.

We generated long-term survival curves and estimated freedom from MACE for patients who underwent three different types of CABG using flexible parametric survival regression (STPM2) with time-varying coefficients (TVC). As a result, the survival difference cannot be determined using the log-rank test. However, the hazard ratios (with p-value and 95%CI) for cardiac-related mortality and MACE were calculated using the same regression method. Additionally, in the supporting information (S2 Fig), we provided a crude analysis of Kaplan-Meier survival and freedom from MACE between the three groups, and we added the p-value for survival differences in the figures.

**Comment 5: Coronary anastomosis is also taking into account sequential grafting or only graft numbers?**

Response: Thank you for pointing this out.

Coronary anastomosis includes the counting from both sequential grafting (side-to-side anastomosis) and end-to-side anastomosis, and it was not account for the number of graft conduits.

**Comment 6: Very interesting results. Re-fine a little bit your propensity matching technique, usually it is well defined for two groups. In the tables the p-values are post hoc corrected? If not, do so to have a realistic number of p-values.**

Response: Thank you for pointing this out.

The p-values were post hoc analyses because the analysis of variance (ANOVA) with Bonferroni was applied for comparisons between groups.

{the separate realistic p-values between each pair, analyzed with ANOVA}

**Comment from reviewer 2**

**Reviewer #2: i read with great interest the MS by Phothikun et al.
the authors concluded OPCAB implementation resulted in a lower occurrence of postoperative ischemic injury than ONCAB and ONBHCAB. No differences in postoperative hemodynamic function in all three techniques were observed. OPCAB respectively were preferable techniques beneficial for long-term outcomes.
the MS is technically sound but i have several comments**

**Comment 1. the authors conducted PS-matching however the process of variables selection is not well defined; i agree n of cases per surgeon should remain adjustment variable but other essential variables (like in Table 1) other than ACEi inhibitors intake should be considered;**

Response: Thank you for pointing this out.

We described in the statistical analysis issue that “The variables included in the model for propensity score were age, sex, New York Heart Association Functional Class, Canadian Cardiovascular Society Classification, the use of angiotensin-converting enzyme inhibitor drugs/angiotensin receptor blockers drugs, the use of pre-operative Aspirin, the pre-operative use of Clopidogrel, end-stage renal disease, coronary artery disease type, and left main coronary disease”. All of these variables were the statistically significant difference when compare between the three groups.

**Comment 2. Authors adjust long-term survival with completeness of revascularization which is correct; why not to PS-match the patients with the n. of anastomoses and/or CR**

Response: Thank you for pointing this out.

We used propensity scores based on the principle that the score refers to the chance of patients being selected into each of the three study groups. Therefore, the variable included in the model should be the variable from the preoperative data or baseline characteristics of the patients. We also think other variables based on perioperative or post-operative data should be used for adjustment in the statistical calculations instead of being included in the propensity score.

**Comment 3. authors mention converted patients were excluded from the study, but how many there were? it seems from the total numbers, OPCAB is not preferred technique (25% of total); are there any factors other than surgeons' preference to drive patients towards one or another approach?**

Response: Thank you for pointing this out.

"62 OPCAB patients (11.9%) were excluded from the study because they were converted to on-CPB CABG. There may have been several factors involved, such as the severity of aortic calcification, stage of kidney disease, LVEF, The severity of coronary stenosis, number of planned anastomosis sites, area of ischemia, and availability of graft conduits. Ultimately, the technique chosen depended on each surgeon's decision."

**Comment 4. OPCAB is further performed predominantly by one surgeon, with higher rates of complete revascularization in this group, contra-intuitively, it poses a limitation; the results of improved survival and reduced ischemia can, therefore, be translatable to broader population of patients only provided that surgeons performing OPCAB have experience in OPCAB and can offer complete revascularization.**

Response: Agree, Thank you for pointing this out.

As this was a retrospective study, the surgeon's experience and skills could have potentially introduced bias in comparative studies of CABG. To address this, we included the surgeon variable as an adjustment variable in our statistical calculations, taking into account differences in the number of CABG techniques used by each surgeon. We used the number of cases performed by each surgeon as a proxy for their experience, which was included in the analysis.

With regard to the completeness of revascularization, our center does not adhere to a strict definition. However, we have a policy of revascularizing all graftable targets in every territory for every case, if feasible, irrespective of techniques. In our study, the mean number of coronary anastomoses performed was 4, which is considered adequate compared to previous studies.

**Comment 5. language editing is required**

Response: Thank you for pointing this out.

In addition to the comments, all spelling and grammatical errors have been corrected again by the staff of the Chiang Mai university English language team (CELT) for language editing assistance.

We look forward to hearing from you in due time regarding our submission and to responding to any further questions and comments you may have.

Sincerely, yours

Amarit Phothikun, MD

Cardiovascular and Thoracic Surgery Unit,
Department of Surgery Faculty of Medicine,
Chiang Mai University, Chiang Mai, Thailand
110 Intravaroros Road, Sriphum, Mueng,
Chiang Mai 50200, Thailand
Email: [armadillos176@gmail.com](mailto:armadillos176@gmail.com) Tel: +66 89 6333 66 1

Thitipong Tepsuwan, MD

Cardiovascular and Thoracic Surgery Unit,
Department of Surgery Faculty of Medicine,
Chiang Mai University, Chiang Mai, Thailand
110 Intravaroros Road, Sriphum, Mueng,
Chiang Mai 50200, Thailand
Email: tepsuwanthitipong@gmail.com Tel: +66815956287
