# Supplementary material for: The outcomes of three different techniques of coronary artery bypass grafting: On-pump arrested heart, on-pump beating heart, and off-pump
Source: PLoS One. 2023 May 31;18(5):e0286510. doi: 10.1371/journal.pone.0286510 (PMC10231783; doi:10.1371/journal.pone.0286510)
Supplement: Attachment — Submitted filename: Response to reviewer ver2.docx [file pone.0286510.s008.docx]

A rebuttal letter.

Dear Vincenzo Lionetti, Acedemic Editor

Thank you for giving us the opportunity to re-submit a second revised draft of our manuscript titled “Outcomes of three different techniques of coronary artery bypass grafting: on-pump arrested heart, on-pump beating heart, and off-pump” to PLOS ONE. We appreciate the time and effort you and the reviewers have dedicated to providing valuable feedback on our manuscript. We are grateful to the reviewers for their insightful comments on our paper. We have been able to incorporate changes to reflect most of the suggestions provided by the reviewers.

Here is a point-by-point response to the academic editor’s and the reviewer’s comments and concerns.

**ACADEMIC EDITOR: The authors should add a new paragraph of discussion regarding perspectives on new early biomarkers of outcome in CABG patients. Recent study has demonstrated that plasma exosome levels increase at 3h and 72h after aortic clamping in older patients undergoing first-time on-pump coronary artery bypass graft and play an antiapoptotic role. In particular, troponin levels did not increase at 72h early after CABG (please see Geroscience 2021 Apr;43(2):773-789). These findings suggest the role of plasma exosomes as perioperative biomarker of tolerance against the perioperative ischemic insult of the heart in CABG patients, as demonstrated by other study (please see PLoS One. 2016 Apr 29;11(4):e0154274.; Acta Anaesthesiol Scand. 2019 Apr;63(4):483-492.) . Please add and discuss the abovementioned studies.**

**Response: Thank you for pointing this out.**

We add a new paragraph of discussion regarding perspectives on “exosome” outcomes in CABG as you recommend. The reference was also added as the same recommendation.

The passage below was the added new passage to the discussion issue.

“In recent times, new biomarkers have emerged that can be compared to cardiac troponin, the current 'gold standard' surrogate biomarker of myocardial damage. Plasma exosomes are small extracellular vesicles that are released into the bloodstream and contain various molecular cargoes that play a role in intercellular communication. During cardiac surgery, ischemia-reperfusion injury can lead to tissue damage and inflammation, resulting in exosome release from injured or stressed cells [16]. Emanueli et al. studied exosomes containing cardiac microRNAs and found that plasma concentrations of exosomes and their cargo of cardiac microRNAs increased in patients undergoing CABG and correlated with cardiac troponin levels [17]. Carrozzo et al. studied between plasma exosomes and serum cardiac troponin I levels in older (on-CPB) CABG patients. After aortic de-clamping, exosome levels significantly increased at both 3 hours and 72 hours, while troponin I level peaked at 3 hours and then gradually decreased [18]. Moreover, Frey et al. discovered that exosome release was related to ischemic/reperfusion injury and associated with myocardial tissue protection [19]. Therefore, due to their persistence and specific biomarkers for cardiac injury, exosomes have the potential to be a valuable new standard tool in the detection and monitoring of myocardial injury in the near future.”

**Comments to the Author**

**Comment from Reviewer 1**

**Reviewer #1:**

**Comment 4. Have the authors made all data underlying the findings in their manuscript fully available?**

**Reviewer #1: No**

**Response: Thank you for pointing this out.**

The new shared data set (data set ver.2) was the data before we had done the propensity matching. We shared the supplyment1 Data “The minimal data set used for analyzed” with the data availability statement: All relevant data are within the paper and its Supporting Information files. Moreover, the new minimal data set can access via; https://doi.org/10.6084/m9.figshare.22723688.v1

**Comment 6. Review Comments to the Author**

**Reviewer #1: Comments are answered properly, please do make available de-identified data (see plos one data policy).**

**Response: Thank you for pointing this out.**

We made available de-identified data in the new shared data set (data set ver.2).

We look forward to hearing from you in due time regarding our re-submission and to respond to any further questions and comments you may have.

Sincerely, yours

Amarit Phothikun, MD

Cardiovascular and Thoracic Surgery Unit,
Department of Surgery Faculty of Medicine,
Chiang Mai University, Chiang Mai, Thailand
110 Intravaroros Road, Sriphum, Mueng,
Chiang Mai 50200, Thailand
Email: armadillos176@gmail.com Tel: +66 89 6333 66 1

Thitipong Tepsuwan, MD

Cardiovascular and Thoracic Surgery Unit,
Department of Surgery Faculty of Medicine,
Chiang Mai University, Chiang Mai, Thailand
110 Intravaroros Road, Sriphum, Mueng,
Chiang Mai 50200, Thailand
Email: tepsuwanthitipong@gmail.com Tel: +66815956287
